# Gynecologic problems and healthcare behavior by shift patterns in Korean nursing staff

**DOI:** 10.1371/journal.pone.0276282

**Published:** 2022-11-01

**Authors:** Miseon Kim, Ju-Hyun Kim, Yong Wook Jung, Seok Ju Seong, Seon-young Kim, Hee-Ja Yoon, Seung-shin Lee, Hyun-Ju Kim, Boon-sun Ku, Hwa-yeon Cho

**Affiliations:** 1 Department of Obstetrics and Gynecology, HM Hospital, Ulsan, Korea; 2 Department of Obstetrics and Gynecology, CHA Gangnam Medical Center, CHA University School of Medicine, Seoul, Korea; 3 Department of Obstetrics and Gynecology, University of Ulsan College of Medicine, Asan Medical Center, Seoul, Korea; 4 Department of Nursing, CHA Gangnam Medical Center, Seoul, Korea; 5 Department of Nursing, CHA Bundang Medical Center, Seongnam, Korea; 6 Department of Nursing, CHA Ilsan Medical Center, Goyang, Korea; 7 Department of Nursing, CHA Gumi Medical Center, Gumi, Korea; 8 Department of Nursing, CHA Seoul Fertility Center, Seoul, Korea; Kyung Hee University School of Medicine, REPUBLIC OF KOREA

## Abstract

We aimed to evaluate the clinical impact of shift patterns at work on gynecologic problems and the healthcare behavior of Korean nursing staff. We conducted a web-based survey for over two weeks in September 2021, involving female nursing staff, including nurses, nurses’ aides, and nursing assistants, working in five medical centers. The questionnaire included 40 questions on baseline characteristics, working information, and experiences with gynecologic problems and medical approaches. Overall, 885 nursing staffs participated in the survey, of the 1,904 who received the invitation with an online link of the survey. The response rate was 46.5%. Among the participants responding to all questions, 569 (64.4%) worked two or three shifts and 305 (34.5%) worked full-time. In women rotating two or three-shift patterns, irregular menstrual cycles (21.6% vs. 13.8%, p = 0.005), abnormal menstrual cycles (40.9% vs. 33.8%, p = 0.038), and dysmenorrhea (48.0% vs. 38.4%, p = 0.006) were more frequent than in those who worked full-time. The experience of visiting gynecologic clinics (47.5% vs. 44.1%, p = 0.332) and treating gynecologic conditions (33.4% vs. 29.3%, p = 0.211) did not differ according to the working patterns. However, diagnosis of gynecologic diseases was more frequent in women working full-time (36.4% vs. 29.7%, p = 0.043). The rates of screening (76.1% vs. 57.8%, p<0.001) and human papillomavirus vaccination (55.7% vs. 39.9%, p<0.001) for cervical cancer prevention were higher in women working full-time than in two–three shifts. This study showed that rotating shift work may be related to an increase in gynecologic problems and a decrease in cancer prevention activity among female nursing staff.

## Introduction

Nursing work inevitably involves rotating shifts owing to the characteristics of the workplace and the nature of work. According to a survey of 173 hospitals with more than 150 beds in South Korea, about 91% of all nurses worked in shifts, with 89.4% working in three shifts [1]. Shift work, including at night, is one of the most common reasons for disrupting the circadian rhythm, which can cause significant changes in sleep and biological function, affecting physical and psychological conditions, and negatively impacting work performance [2–4]. It has not been clearly concluded yet how shift work affects reproductive health, although women account for more than 90% of nurses [5].

Various environmental stressors, such as stress related to daily life and work, can interfere with endocrine function and cause menstrual disorders, which are indicators of reproductive health [6–9]. The hypothalamus–pituitary–ovarian axis regulates the cyclic secretion of reproductive hormones, including estrogen, progesterone, luteinizing hormone, and follicle-stimulating hormone, controlling menstruation [10]. Any disturbance of the circadian rhythm during shift work influences the balance of the hypothalamus–pituitary–ovarian axis and induces menstrual disorders [11,12].

However, there has been still controversy so far regarding the association between shift work and gynecologic symptoms, including menstrual disorders [13–15]. Moreover, there is no domestic research on the correlation between pattern of work and gynecologic healthcare behavior in nursing staffs in Korea. This study aims to compare the gynecologic symptoms, visits to the gynecologic clinic, and cancer prevention activities of female nurses working full-time and having shift patterns at work using a web-based survey.

## Materials and methods

### Study design and population

A web-based survey was conducted from September 19–24, 2021. The online invitation to assess the questionnaire was sent to 1,904 nursing staff working at five medical centers. Of the 1,904 participants who received the invitation, 885 voluntarily participated in the survey. The survey was conducted with nursing staff, including female nurses, nurses’ aides, and nursing assistants. The study protocol was approved by the Institutional Review Board of CHA Gangnam Medical Center (IRB No. 2021-06-008-002). All participants gave informed consent before taking part in this study.

### Questionnaire

All the participants responded to 40 questions using an online questionnaire (S1 Table). The questionnaire consisted of socio-demographic information (11 questions), work information (11 questions), and experiences of gynecologic problems and medical approaches in the last two years (18 questions). Socio-demographic information included age, height, weight, age at menarche, marital status, parity, smoking, and coffee consumption. We investigated the duration of work, the department in which the participant was working, shift patterns, night shift status, customer service status, average time spent standing, and time spent working on a computer. We also assessed exposure to chemicals, including anesthetics, anti-cancer drugs, antiseptics, and formaldehyde, at the workplace.

Gynecologic problems experienced in the past two years included irregular menstrual cycle, abnormal menstrual period, abnormal menstrual volume, and dysmenorrhea. An irregular menstrual cycle was defined as menstruation with an interval of <21 days or >35 days. An abnormal menstrual period was defined as <3 days or >7 days [16]. Abnormal menstrual volume was defined as <20 ml or >80 ml. Dysmenorrhea intensity was measured using the Numerical Rating Scale (NRS), and dysmenorrhea >NRS 4 was considered severe dysmenorrhea in this study [17,18]. Abnormal uterine bleeding was defined as incidental bleeding outside the routine menstrual period [19].

### Statistics

Descriptive statistics were used to analyze the characteristics and working information of the study participants. To determine whether there was a correlation between variables, we used the chi-square test for categorical variables with a p-value of <0.05 considered as statistically significant. Logistic regression was used to identify the independent factors associated with a long lead time to colposcopy using the Pap test. Variables with p-value of <0.25 were included in the multivariate analysis, and P < 05 was considered to be significant. IBM SPSS Statistics for Windows, version 24.0 (IBM Corp., Armonk, NY, USA) was used for all statistical analyses. We followed the statistical guidelines [20,21].

## Results

### Baseline characteristics and working information

The socio-demographic characteristics of the participants and information on the working conditions of the respondents (n = 885) are presented in Table 1. The median age of the nursing staff was 31 (range: 23‒63). The median age at menarche was 13 (range, 9‒19 years). A total of 502 (56.7%) were unmarried and 301 (34.0%) had never given birth. Seventy (7.9%) women had experienced menopause. Among the participants, 852 (96.3%) had never smoked; 169 (19.1%) participants did not drink coffee; however, 60 (6.8%) drank more than three cups of coffee per day. Two hundred and four (23.1%) had less than 2 years, 394 (44.5%) had 2–9 years, 209 (23.6%) had 10–19 years, and 78 (8.8%) had over 20 years of work experience. Most of the participants worked in the general ward (43.1%), followed by the outpatient clinic (19.5%), and the operating room (15.4%). Regarding shift patterns, 503 women (56.9%) worked three shifts, 305 (34.5%) worked full time, and 66 (7.5%) worked two shifts. Among the participants, 766 (86.6%) served customers, 476 (53.8%) used a computer for >6 hours a day, and 385 (43.5%) worked in a standing position for >6 hours a day.

**Table 1:** Baseline characteristics and working information (n = 885).

Characteristic	Value
Age, yrs	31 (23‒63)
Height, cm	161 (146‒175)
Weight, kg	55(38‒99)
Menarche, yrs	13(9‒19)
Married	
No	502 (56.7)
Yes	383 (43.3)
Nulliparous	
No	584 (66.0)
Yes	301 (34.0)
Menopause	
No	815 (92.1)
Yes	70 (7.9)
Smoking	
No	852 (96.3)
Yes, within 1 year	18 (2.0)
Yes, but before 1 year ago	15 (1.7)
Coffee, per day	
No	169 (19.1)
1 cup	429 (48.5)
2 cup	227 (25.6)
3 cup more	60 (6.8)
Career for nurse	
<2 year	204 (23.1)
2‒9 years	394 (44.5)
10‒19 years	209 (23.6)
>20 years	78 (8.8)
Working departments	
Outpatient clinic	173 (19.5)
Wards	381 (43.1)
Operating room	136 (15.4)
Delivery room	40 (4.5)
Emergency room	58 (6.6)
Others	97 (11.0)
Shift patterns	
Full-time	305 (34.5)
Two shifts	66 (7.5)
Three shifts	503 (56.9)
Others	11 (1.2)
Night duty	
None	334 (37.7)
<3	62 (7.0)
4‒5	162 (18.3)
6‒7	241 (27.2)
>8	86 (9.7)
Serve a customer	
No	119 (13.4)
Yes	766 (86.6)
Standing, hours per day	
<2	78 (8.8)
2‒4	193 (21.8)
4‒6	229 (25.9)
>6	385 (43.5)
Watching the computer monitor	
<2	117 (13.2)
2‒4	98 (11.1)
4‒6	194 (22.0)
>6	476 (53.8)

The values are presented as the median (range) or number (%), unless otherwise indicated.

Young, unmarried, and nulliparous women were common in the shift working group. (Table 2) There was no difference in the number of women who served customers between the two working groups (88.2% vs. 85.85%, p = 0.314). However, respondents who used a computer for >6 hours a day were more common in the full-time group (63.9% vs. 48.9%, p<0.001), and those who worked in a standing position >6 hours a day were more common in the shift working group (51.0% vs. 29.8%, p<0.001).

**Table 2:** Comparison of the characteristics by working patterns (n = 874).

Characteristic	Full-time (n = 305)	Two or three shifts (n = 569)	p-value
Age >31 years^a^	221 (72.5)	215 (37.8)	**<0.001**
Height >160 cm^a^	114 (47.2)	292 (51.3)	0.247
Weight >55 kg^a^	139 (45.6)	257 (45.2)	0.908
Menarche >13 years^a^	171 (56.1)	272 (47.8)	**0.020**
Married			**<0.001**
No	121 (39.7)	376 (66.1)	
Yes	184 (60.3)	193 (33.9)	
Nulliparous			**<0.001**
No	153 (50.2)	140 (24.6)	
Yes	152 (49.8)	429 (75.4)	
Menopause			0.584
No	283 (92.8)	522 (91.7)	
Yes	22 (7.2)	47 (8.3)	
Current or past smoking			**<0.001**
No	304 (99.7)	538 (94.6)	
Yes	1 (0.3)	31 (5.4)	
Coffee ≥3 cup per day			**0.036**
No	277 (90.8)	538 (94.6)	
Yes	28 (9.2)	31 (5.4)	
Career for nurse ≥10 years			**<0.001**
No	143 (46.9)	450 (79.1)	
Yes	162 (53.1)	119 (20.9)	
Serve a customer			0.314
No	36 (11.8)	81 (14.2)	
Yes	269 (88.2)	488 (85.8)	
Standing >6 hours per day			**<0.001**
No	214 (70.2)	279 (49.0)	
Yes	91 (29.8)	290 (51.0)	
Watching the computer monitor >6 hours per day			**<0.001**
No	110 (36.1)	291 (51.1)	
Yes	195 (63.9)	278 (48.9)	

The values are presented as the median (range) or number (%), unless otherwise indicated.

^a^Median value.

### Menstrual disorders

**Fig 1:**
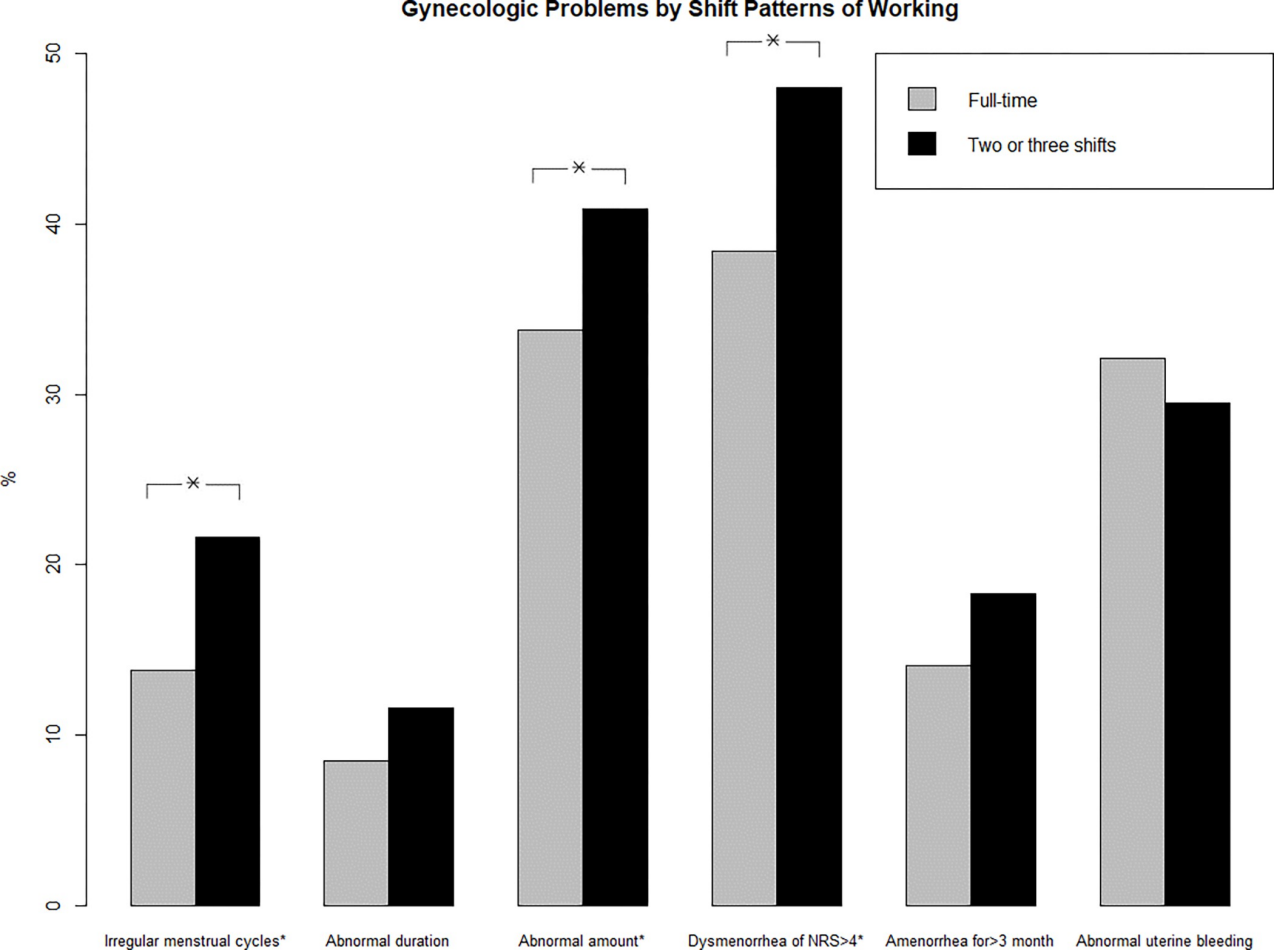
Gynecologic problems by shift patterns of working.

Menstrual problems in the last two years according to the type of work are presented in Fig 1. A statistically significant increase in the number of irregular menstrual cycles (21.6% vs. 13.8%, p = 0.005) and abnormal menstrual cycles (40.9% vs. 33.8%, p = 0.038) was observed in those who worked two or three shifts compared with full-time workers. Participants who worked two or three shifts also had more dysmenorrhea, with an NRS score of >4 (48.0% vs. 38.4%, p = 0.006). The proportion of women experiencing abnormal menstrual duration (8.5% vs. 11.7%, p = 0.142), amenorrhea >3 months (14.1% vs. 17.9%, p = 0.145), and abnormal uterine bleeding (32.1% vs. 29.7%, p = 0.447) did not differ. In contrast, exposure to chemicals, including anesthetics, chemoagents, disinfectants, and formaldehyde, did not increase menstrual problems (S2 Table).

Women who experience ≥2 abnormal menstrual symptoms were 411/874 (47.0%) in this study. (Table 3) Shift working (Odd ratio [OR] = 1.439, 95% confidence interval [CI] = 1.056‒1.959; p = 0.021) and history of smoking (OR = 2.322, 95% CI = 1.047‒5.147; p = 0.038) are independent adverse factors for the ≥2 abnormal menstrual symptoms in the multivariate analysis.

**Table 3:** Factors associated with experiences of 2 and more gynecologic symptoms^a^.

Characteristic	Univariate		Multivariate	
	OR (95% CI)	p	OR (95% CI)	p
Age ≤31 years^b^	1.878 (1.435–2.458)	**<0.001**	1.359 (0.879–2.100)	0.167
Menarche ≤13 years^b^	0.918 (0.703–1.197)	0.526	-	-
Unmarried	1.739 (1.325–2.282)	**<0.001**	1.156 (0.710–1.882)	0.561
Nulliparous	1.751 (1.314–2.332)	**<0.001**	1.109 (0.663–1.854)	0.694
Current or past smoking	2.990 (1.367–6.539)	**0.006**	2.322 (1.047–5.147)	**0.038**
Coffee ≥3 cup per day	1.267 (0.747–2.151)	0.380	-	-
Career for nurse <10 years	1.748 (1.308–2.337)	**<0.001**	1.012 (0.667–1.535)	0.955
Standing >6 hours per day	1.370 (1.047–1.792)	**0.022**	1.177 (0.886–1.562)	0.261
Watching the computer monitor >6 hours per day	1.011 (0.774–1.319)	0.938	-	-
Shift working	1.829 (1.377–2.431)	**<0.001**	1.439 (1.056–1.959)	**0.021**

The values are presented as the median (range) or number (%), unless otherwise indicated.

OR, odd ratio; CI, confidence interval.

^a^Among irregular menstrual cycles, abnormal menstrual duration, abnormal menstrual amount, dysmenorrhea >4 NRS, amenorrhea >3 months, abnormal uterine bleeding.

^b^Median value.

In stratified women who worked two or three shifts, women with amenorrhea >3 months were more frequently found in the group that did not serve customers (27.2% vs. 16.8%, p = 0.026). However, there was no difference in the rate of women with irregular cycles (22.3% vs. 17.3%, p = 0.306), abnormal duration (10.9% vs. 16.0%, p = 0.177), abnormal volume (40.2% vs. 45.7%, p = 0.350), and dysmenorrhea (49.0% vs. 42.0%, p = 0.243), irrespective of whether or not they served customers. Women who worked in a standing position for >6 hours a day expressed dysmenorrhea with NRS >4 (52.1% vs. 43.7%, p = 0.046) and amenorrhea >3 months (22.8% vs. 13.6%, p = 0.005). The use of a computer for >6 hours did not affect menstrual symptoms in stratified women who worked two or three shifts (data not shown), respectively.

### Healthcare behavior

The experiences of diagnosis and treatment for gynecological diseases were compared between women working full-time and in two or three shifts (Table 4). The proportion of women visiting the outpatient clinic due to gynecologic problems did not differ between the two groups (47.5% vs. 44.1%, p = 0.332). More women with full-time work diagnosed gynecologic disease (36.4% vs. 29.7%, p = 0.043); however, the treatment experiences were similar to those of women working in two or three shifts (33.4% vs. 29.3%, p = 0.211). The percentage of respondents who received regular Pap tests every three years (76.1% vs. 57.8%, p<0.001) and completely received human papillomavirus (HPV) vaccination (55.7% vs. 39.9%, p<0.001) was higher in women working full-time than in those working in two or three shifts. Among all responders, 365 women (41.2%) underwent Pap tests by the national screening program, and 243 (27.5%) received the private Pap test. Alternatively, 235 (26.6%) patients did not undergo Pap tests. When asked what was the biggest barrier in receiving Pap tests, 314 women (35.5%) answered no time, 300 (33.9%) said the inconvenience and discomfort from the Pap test itself, 158 (17.9%) missed the timing of Pap tests, and 52 (5.9%) said that they lacked awareness of the need for Pap tests. Furthermore, 457 (51.6%) were not vaccinated for HPV, 403 (45.5%) completed HPV vaccination, and 25 were currently vaccinated. Meanwhile, 498 women (56.2%) believed that high cost was the greatest barrier to completing the HPV vaccination, followed by a lengthy vaccination schedule lasting six months (n = 156, 17.6%), lack of awareness of the need (n = 145, 16.4%), and concerns about side effects (n = 69, 7.8%) (data not shown in the table).

**Table 4:** Experiences of medical approach and screening for gynecologic disease by workingpatterns (n = 874).

Characteristic	Full-time (n = 305)	Two or three shifts (n = 569)	p-value
Taking oral pill			0.185
No	147 (48.2)	301 (52.9)	
Yes	158 (51.8)	268 (47.1)	
Visiting the gynecologic clinic			0.332
No	160 (52.5)	318 (55.9)	
Yes	145 (47.5)	251 (44.1)	
Recommended the exam by doctor			0.236
No	197 (64.6)	390 (68.5)	
Yes	108 (35.4)	179 (31.5)	
Diagnosed the gynecologic disease			**0.043**
No	194 (63.6)	400 (70.3)	
Yes	111 (36.4)	169 (29.7)	
Treated the gynecologic disease			0.211
No	203 (66.6)	402 (70.7)	
Yes	102 (33.4)	167 (29.3)	
Regular Pap test per 3 years			**<0.001**
No	73 (23.9)	240 (42.2)	
Yes	232 (76.1)	329 (57.8)	
Complete vaccination for HPV			**<0.001**
No	135 (44.3)	342 (60.1)	
Yes	170 (55.7)	227 (39.9)	

Pap test, papanicolaou test; HPV, human papillomavirus.

## Discussion

In this study, we attempted to determine the association between various occupational hazards and gynecologic problems among nursing staff in Korea. Women working two or three shifts had more menstrual irregularities, abnormal menstrual volume, and dysmenorrhea than women working full time. The medical approaches to gynecologic problems were similar between the two groups. Although there were more women diagnosed with gynecologic disease in the full-time work group, there was no difference in the rates of women treated for the disease. Notably, full-time workers had significantly higher cervical cancer screening and HPV vaccination rates than shift workers.

This finding is similar to previous studies. Labyak et al. [22] reported that 53% of nurses with shift work experienced menstrual changes among 68 nurses under 40 years of age. The authors suggested that women who experienced menstrual changes had more physiological symptoms, and sleep disturbances may lead to menstrual irregularities. According to a study by Jiang et al. [14] 41% of 8,904 nurses surveyed had experienced menstrual disorders and night work was also moderately related with abnormal menstrual duration (OR = 1. 24, 95% CI = 1.04–1.48) and irregular menstrual cycles (OR = 1.20, 95% CI = 1.06–1.35) [14]. Due to disruption of the circadian rhythm during shift work, the imbalance of the hypothalamic-pituitary-ovarian axis can cause menstrual problems. [11,12]. Jiang et al. showed that the use of disinfectants on duty was the biggest risk factor for menstrual disorders (OR = 1.53, 95% CI = 1.39–1.68) [14]. In our study, the use of anesthetics, chemoagents, disinfectants, and formaldehyde was not associated with menstrual problems, in contrast to Jiang’s study. This discrepancy can be attributed to differences in the policy for handling the chemicals, the types of chemicals, or the criteria for menstrual problems between the two studies. There is limited evidence on the relationship between human reproductive data and chemicals [23].

Nevertheless, the debate continues regarding the association between menstrual problems and working shift patterns. A study of 1,249 nurses in 2020 reported that working in two or three rotating shifts increased the incidence of irregular menstrual cycles compared to no night shifts; however, the incidence of dysmenorrhea affecting their work was not significantly different according to shift patterns (p = 0.429) [15]. A recent prospective epidemiological study of 188 Spanish nurses revealed that rotating shift work patterns did not increase the risk of menstrual disorders, including prolonged duration, dysmenorrhea, prolonged duration of dysmenorrhea, and excessive bleeding [13]. Regarding the impact of night shift work on women’s reproductive health, an integrated literature review of 20 articles by Chau et al. [5] determined that the evidence was insufficient to draw conclusions and that further study was needed. In our study, abnormal duration, amenorrhea for >3 months, and abnormal uterine bleeding were not different between women working full-time and in shifts, unlike menstrual irregularities, abnormal menstrual volume, and dysmenorrhea.

Women working full-time more frequently visited the gynecologic clinic (not statistically significant), recommended the examination (not statistically significant), and diagnosed the disease (p = 0.043) in this study. Most women working full-time in this study had worked in outpatient departments; therefore, they may be more likely to have high accessibility to gynecology clinics in their workplace. Similarly, the proportions of regular Pap tests and completion of the HPV vaccination were higher in full-time nurses than in those working in two or three shifts. The reason for this may be that full-time workers have more day time activities. However, even the rate of the Pap test in women’s work shifts (57.8%) is not inferior to that of the general population (54.8% in 2020 reported by Statistics Korea), and their rate of HPV vaccine uptake (39.9%) is higher than that of the general population (14.2%) [24]. We must exercise caution while interpreting this result because the working pattern of nurses usually fluctuates in the short term. We surveyed the experiences within two years and the last working pattern; however, the working pattern may change in the short term for less than two years.

Our study has certain limitations. It had a recall bias owing to self-reported measures and the low response rate could lead to sampling bias. Therefore we cannot extrapolate our results to the entire nurses. Additionally, we did not investigate whether the respondents were currently being treated for gynecologic diseases or whether they were taking hormonal drugs. Owing to the nature of this study, we could not verify the medical records of the participants. The gynecologic diseases mentioned by the participants may not be considered as actual diseases requiring treatment. Nevertheless, this study is the first large-scale survey in Korea that attempts to demonstrate the association between working patterns and gynecologic problems, including healthcare behavior, in addition to stress intensity or working performance.

In summary, rotating shift patterns in nursing staff were related to irregular menstrual cycles, abnormal menstrual cycles, and more intense dysmenorrhea. Moreover, shift nursing staff had lower cervical cancer screening and HPV vaccination rates than full-time workers. The rotating shift work pattern may be associated with gynecologic symptoms and healthcare behavior of nursing staff.

## Supporting information

S1 Table Questionnaire in this study. (DOCX)

S2 Table Gynecologic problems by handling chemicals (n = 885). (DOCX)
