# Supplementary material for: Gynecologic problems and healthcare behavior by shift patterns in Korean nursing staff
Source: PLoS One. 2022 Nov 1;17(11):e0276282. doi: 10.1371/journal.pone.0276282 (PMC9624425; doi:10.1371/journal.pone.0276282)
Supplement: S1 Table — (DOCX) [file pone.0276282.s001.docx]

Gynecologic problems and healthcare behavior by shift patterns in Korean nursing staff

Miseon Kim^1,^ Ju-Hyun Kim^2*^, Yong Wook Jung^3^, Seok Ju Seong^3^, Seon-young Kim^4^, Hee-Ja Yoon^4^, Seung-shin Lee^5^, Hyun-Ju Kim^6^, Boon-sun Ku^7^, Hwa-yeon Cho^8^

^1^Department of Obstetrics and Gynecology, HM hospital, Ulsan, Korea

^2^Department of Obstetrics and Gynecology, University of Ulsan College of Medicine, Asan Medical Center, Seoul, Korea

^3^Department of Obstetrics and Gynecology, CHA Gangnam Medical Center, CHA University School of Medicine, Seoul, Korea

^4^Department of Nursing, CHA Gangnam Medical Center, Seoul, Korea

^5^Department of Nursing, CHA Bundang Medical Center, Seongnam, Korea

^6^Department of Nursing, CHA Ilsan Medical Center, Goyang, Korea

^7^Department of Nursing, CHA Gumi Medical Center, Gumi, Korea

^8^Department of Nursing, CHA Seoul Fertility Center, Seoul, Korea

*Corresponding author:

E-mail: smilekako@naver.com (JHK)

**S1 Table.** **Questionnaire in This Study.**

**Questionnaire about gynecological symptoms and medical care according to type of work**

*Hello,*

*We want to survey the gynecological symptoms and medical consultation according to type of work among the nursing division staff. The questionnaire will take about three minutes, and we ask that you answer all of the questions. The responses will only be used for research purposes, and personal information will strictly be protected. There will be no disadvantages for participating in the survey.*

*We appreciate your participation.*

1. Do you agree to participate in the survey?

□ Yes □ No

1. What is your year of birth? (Only numbers)
2. Height ( ) cm
3. Weight ( ) kg
4. What age did you have your first period? ( ) years
5. What is your marital status?

□ Married □ Single □Cohabitation□ Widowed

1. Have you given birth?

□ Yes □ No

1. How many children do you currently have?

- ≥3 □ 2 □ 1 □ None

1. Have you had menopause?

□ Yes □ No

1. What is your smoking history or current smoking status?

- I have smoked a cigarette within the past year.
- I used to be a smoker but quit more than a year ago.
- I have never been a smoker.

1. How many cups of coffee do you have a day on average?

- ≥3 cups □ 2 cups □ 1 cup □None

***Work in the past two years: answer based on your work in the past two years, and if you had a transfer, answer based on your primary place of work.***

1. How many years have you worked?

□ ≥ 20 years □ 10–19 years □ 2–9 years □ 2 years

1. Where do you work? (Ob &Gynincludes obstetrics, gynecology, and infertility-related departments, and if other, specify the department.)

□ Outpatient Ob &Gyn clinic, Ob &Gyn ward (more than half of the patients are Ob &Gyn patients)

□ Ob &Gyn operation room (where Ob &Gyn operation is more than 50%)

□ Delivery room

□ Emergency department

□ Other:

1. What is your work schedule? (If other, specify.)

□Full time (daytime, conventionally 8 am–5 pm)

□ 12-hour rotating shift

□ 8-hour rotating shift

□ Other:

1. If you work night shifts, how many night shifts do you work per month?

□ ≥8 □ 6–7 □ 4–5 □ ≤3 □ none

1. Do you deal with patients or consult patients at your workplace?

□ Yes □ No

1. How many hours a day are you on your feet on average?

□ ≥6 hours □ 4–6 hours □ 2–3 hour □<2 hours

1. How many hours a day are you in front of a computer on average?

□ ≥6 hours □ 4–6 hours □ 2–3 hour □<2 hours

1. Do you handle anesthetics?

□ Yes □ No

1. Do you handle anticancer agents?

□ Yes □ No

1. Do you handle equipment disinfectants?

□ Yes □ No

1. Do you handle formaldehyde (formalin)?

□ Yes □ No

***Gynecological symptoms in the past two years: answer based on the past two years***

1. Do you have a regular menstrual cycle?

□ Yes □ No

1. What is your average menstrual cycle?

□<21 days □ 21–35 days □>35 days

1. How many days of menstrual bleeding do you have on average?

□<3 days □ 3–7 days □>7 days

1. How heavy is your average menstrual flow throughout the entire menstrual period?

□<20ml (About a table spoon)

□ 20‒60ml

□>60ml (fully filled a small cup)

1. How intense is your menstrual pain? ( )

(NRS, Numeral Rating Scale: 10 for so severe to hinder daily life, 0 to 10)

1. Have you ever skipped your menstrual period for more than three times your normal menstrual cycle or more than three months?

□ Yes □ No

1. Have you ever had abnormal uterine bleeding beyond your normal menstrual period?

□ Yes □ No

1. Have you ever taken female hormone drugs? (If other, specify.)

□ Never

□ I’ve tried them for contraceptive purposes.

□ I’ve tried them to control gynecological symptoms such as menstrual pain and abnormal bleeding.

□ I’ve tried them to alter my menstrual cycle before traveling or important events.

□ I’ve tried them to treat a gynecological disease.

□ I’ve tried them to prevent gynecological cancer.

□ Other:

***Ob &Gyn care in the past 2 years***

1. Have you seen an Ob &Gyn specialist for gynecological symptoms (not regular check-up)?

□ Yes □ No

1. Have you ever been referred for testing by an Ob &Gyn specialist?

□ I’ve been referred for ultrasound.

□ I’ve been referred for abdomen/pelvis CT.

□ I’ve been referred for a blood test.

□ I’ve been referred for a biopsy.

1. Have you ever been diagnosed with an obstetric/gynecological disease?

□ Yes □ No

1. Have you ever been treated for an obstetric/gynecological disease?

□ No

□ I’ve had pharmacological treatment.

□ I’ve had a procedure or surgery.

□ Other:

1. Do you usually see an Ob &Gyn doctor at your workplace?

□ Yes □ No

1. Have you had a pap smear in the past three years?

□ Yes □ No

1. Do you undergo the biennial Pap test through the national screening program offered by the National Health Insurance Service (NHIS)?

□ I utilize the national screening program every time.

□ I don’t utilize it every time but I have utilized it once.

□ I don’t utilize the national screening program but have a pap smear periodically on my own.

□ I have never had a pap smear.

1. What do you think is the greatest barrier to having a periodic pap smear?

□ Lack of time

□ Fear of abnormal results

□Missing the timing of the test

□ Inconvenience and discomfort from the test itself

□ Lack of awareness of the need

□ Other:

1. Have you completed all three doses of the human papillomavirus (HPV) vaccine for cervical cancer prevention?

□ Yes □ I’m currently receiving my doses□ No

1. What do you think is the greatest barrier to completing the HPV vaccination?

□ High cost

□ Long vaccination schedule lasting 6 months

□ Concerns about side effects

□ Lack of awareness of the need

□ Other:
