# Supplementary material for: Gynecologic problems and healthcare behavior by shift patterns in Korean nursing staff
Source: PLoS One. 2022 Nov 1;17(11):e0276282. doi: 10.1371/journal.pone.0276282 (PMC9624425; doi:10.1371/journal.pone.0276282)
Supplement: S2 Table — (DOCX) [file pone.0276282.s002.docx]

Gynecologic problems and healthcare behavior by shift patterns in Korean nursing staff

Miseon Kim^1,^ Ju-Hyun Kim^2*^, Yong Wook Jung^3^, Seok Ju Seong^3^, Seon-young Kim^4^, Hee-Ja Yoon^4^, Seung-shin Lee^5^, Hyun-Ju Kim^6^, Boon-sun Ku^7^, Hwa-yeon Cho^8^

^1^Department of Obstetrics and Gynecology, HM hospital, Ulsan, Korea

^2^Department of Obstetrics and Gynecology, University of Ulsan College of Medicine, Asan Medical Center, Seoul, Korea

^3^Department of Obstetrics and Gynecology, CHA Gangnam Medical Center, CHA University School of Medicine, Seoul, Korea

^4^Department of Nursing, CHA Gangnam Medical Center, Seoul, Korea

^5^Department of Nursing, CHA Bundang Medical Center, Seongnam, Korea

^6^Department of Nursing, CHA Ilsan Medical Center, Goyang, Korea

^7^Department of Nursing, CHA Gumi Medical Center, Gumi, Korea

^8^Department of Nursing, CHA Seoul Fertility Center, Seoul, Korea

*Corresponding author:

E-mail: smilekako@naver.com (JHK)

**S2 Table. Gynecologic Problems by Handling Chemicals (n=885).**

|  | **Total** | **Irregular cycles** | **Abnormal duration** | **Abnormal amount** | **Dysmenorrhea** |
| --- | --- | --- | --- | --- | --- |
| Anesthetics |  |  |  |  |  |
| No | 684 (77.3) | 125 (18.3) | 74 (10.8) | 251 (36.7) | 295 (43.1) |
| Yes | 201 (22.7) | 42 (20.9) | 20 (10.0) | 88 (43.8) | 102 (50.7) |
| p-value |  | 0.404 | 0.725 | 0.069 | 0.056 |
| Chemoagents |  |  |  |  |  |
| No | 758 (85.6) | 139 (18.3) | 85 (11.2) | 285 (37.6) | 343 (45.3) |
| Yes | 127 (14.4) | 28 (22.0) | 9 (7.1) | 54 (42.5) | 54 (42.5) |
| p-value |  | 0.323 | 0.162 | 0.291 | 0.567 |
| Disinfectants |  |  |  |  |  |
| No | 385 (43.5) | 85 (22.0) | 49 (12.7) | 147 (38.2) | 171 (44.4) |
| Yes | 500 (56.5) | 82 (16.4) | 45 (9) | 192 (38.4) | 226 (45.2) |
| p-value |  | **0.032** | 0.074 | 0.947 | 0.816 |
| Formaldehyde |  |  |  |  |  |
| No | 662 (74.8) | 130 (19.6) | 69 (10.4) | 263 (39.7) | 292 (44.1) |
| Yes | 223 (25.2) | 37 (16.6) | 25 (11.2) | 76 (34.1) | 105 (47.1) |
| p-value |  | 0.315 | 0.741 | 0.134 | 0.440 |

The values are presented as number (%).
